# Effect of Progressive Fatigue on Session RPE

**DOI:** 10.3390/jfmk5010015

**Published:** 2020-02-17

**Authors:** Andrea Fusco, William Sustercich, Keegan Edgerton, Cristina Cortis, Salvador J. Jaime, Richard P. Mikat, John P. Porcari, Carl Foster

**Affiliations:** 1 Department of Human Sciences, Society and Health, University of Cassino and Lazio Meridionale, 03043 Cassino, Italy; Italy; c.cortis@unicas.it; 2 Department of Exercise and Sport Science, University of Wisconsin-La Crosse, La Crosse, WI 54601, USA; wssustercich@gmail.com (W.S.); keegan.edgerton@irhythmtech.com (K.E.); sjaime@uwlax.edu (S.J.J.); rmikat@uwlax.edu (R.P.M.); jporcari@uwlax.edu (J.P.P.); cfoster@uwlax.edu (C.F.)

**Keywords:** overtraining, training load, overreaching, performance, glycogen

## Abstract

Rating of perceived exertion (RPE) and session RPE (sRPE) are reliable tools for predicting exercise intensity and are alternatives to more technological and physiological measurements, such as blood lactate (HLa) concentration, oxygen consumption and heart rate (HR). As sRPE may also convey some insights into accumulated fatigue, the purpose of this study was to examine the effects of progressive fatigue in response to heavier-than-normal training on sRPE, with absolute training intensity held constant, and determine its validity as marker of fatigue. Twelve young adults performed eight interval workouts over a two-week period. The percentage of maximal HR (%HRmax), HLa, RPE and sRPE were measured for each session. The HLa/RPE ratio was calculated as an index of fatigue. Multilevel regression analysis showed significant differences for %HRmax (*p* = 0.004), HLa concentration (*p* = 0.0001), RPE (*p* < 0.0001), HLa/RPE ratio (*p* = 0.0002) and sRPE (*p* < 0.0001) across sessions. Non-linear regression analysis revealed a very large negative relationship between HLa/RPE ratio and sRPE (*r* = −0.70, *p* < 0.0001). These results support the hypothesis that sRPE is a sensitive tool that provides information on accumulated fatigue, in addition to training intensity. Exercise scientists without access to HLa measurements may now be able to gain insights into accumulated fatigue during periods of increased training by using sRPE.

## 1. Introduction

It is well known that adaptive responses to training programs are dependent on the frequency, intensity and time of training (e.g., the FIT principle). Training intensity is arguably the most complex aspect of training program design. The careful monitoring of training intensity is useful to maximize performance gains and minimize side-effects, such as non-functional overreaching, injury, and illness [1,2].

Training intensity can be monitored [3] by many objective physiological markers, such as heart rate (HR), oxygen consumption (VO_2_), and blood lactate concentration (HLa). There has been much discussion surrounding which physiological measure may be best for monitoring, in particular, the effects indicative of maladaptive states, such as non-functional overreaching [3]. One frequent observation during periods of heavy training is a markedly reduced maximal HLa concentration, while submaximal values remain unchanged or slightly reduced [4]. Acute increases in training workload can lead to acute glycogen depletion, which has been shown to correlate with decreases in average [5] and maximal [6] HLa concentration. As HLa is a byproduct of glycogenolysis, average and maximal HLa concentrations may be used as a surrogate to estimate decreases in muscle glycogen concentration. High exercise intensities or durations during consecutive training days have been shown to lower muscle glycogen levels and decrease HLa concentration, resulting in fatigue and hindering athletic performance [5,7]. Although monitoring HLa concentrations is potentially useful for estimating glycogen depletion and monitoring training intensity, it is costly and time-consuming, and can only partially account for changes in muscle glycogen. A simpler and more affordable way would be preferred. Less costly and more accessible subjective methods, such as the rating of perceived exertion (RPE) and session RPE (sRPE), have become attractive [8,9,10,11]. Previous research has shown that sRPE is a reliable tool for predicting exercise intensity compared to more direct measurements, such as HR and HLa concentration [9,12,13]. Within the concept of the training impulse (TRIMP) [14] score, individuals are asked, typically thirty minutes after completing a training session, how their workout felt [9,10]. Subsequently, to quantify the exercise training load, sRPE is multiplied by the duration of training. Thus, the sRPE-derived training load may be used as an indicator of internal training load [15]. Recent evidence suggests that sRPE not only provides information related to intensity, but also conveys information about progressive fatigue [16]. In particular, sRPE provides information on accumulated fatigue that is not available from accepted markers of internal training intensity, such as HR and HLa concentration. In our view, both the momentary RPE and sRPE are understood primarily as surrogates of exercise intensity. If exercise intensity is the only use of sRPE, then it should not drift when longer training bouts are used (e.g., increased fatigue). A previous study [16] suggested that sRPE progressively increased during a course of prolonged exercise training (within days) although objective measures of intensity, such as pace, HR and HLa concentration did not change, which was also noted by Foster and colleagues [10]. The present study represents a further exploration of these findings (between days).

Since the process of monitoring training is intended to provide coaches and athletes with information about the entire response to training [1], a better understanding of how sRPE responds under different circumstances would be helpful to optimize the use of this very simple method of training monitoring. Therefore, the purpose of this study was to examine the effects of progressive fatigue that occur in response to heavier-than-normal training on sRPE, with the intent of exploring its potential as a marker of fatigue. The study was designed to test the hypothesis that sRPE for a given exercise bout would increase with progressive fatigue, whether from a longer exercise bout, or from successive days of harder-than-usual bouts. 

## 2. Materials and Methods 

### 2.1. Participants

Twelve physically active (>150 minutes at moderate intensity per week) college-age students (six males; six females) provided written informed consent and completed the protocol approved by the Institutional Review Board for the Protection of Human Subjects of the University of Wisconsin-La Crosse (approval number: 45CFR46; date: 8 September 2016). The subjects were students recruited from the University community, limiting the sample to those who exercised regularly to avoid a large training effect from participation in the protocol.

### 2.2. Procedures

Subjects were familiarized with the Borg Category Ratio (0–10) RPE [11] and the session RPE (sRPE) [9,10] scales before the beginning of the study. Each subject completed a maximal incremental test on an electrically braked cycle ergometer (Lode Excalibur, Groningen, Netherlands) with respiratory gas exchange (AEI Moxus, Pittsburg, PA, USA) to evaluate peak VO_2_ (VO_2peak_), maximal HR (HR_max_) and peak power output (PPO). They were tested >3 h postprandial, had refrained from alcohol consumption and heavy exercise >24 h prior to testing, and abstained from caffeine consumption >6 h prior to testing. The initial power output was 25W and was increased by 25W every 2 min until volitional fatigue. Subjects were instructed to maintain a pedaling rate of 60–80 revolutions per minute. Subsequently, the subjects completed thirty-minute and sixty-minute interval workouts on the same electrically braked cycle ergometer. The cycle ergometer was chosen for convenience with the measurements and for controlling the workload.

**Figure 1:**
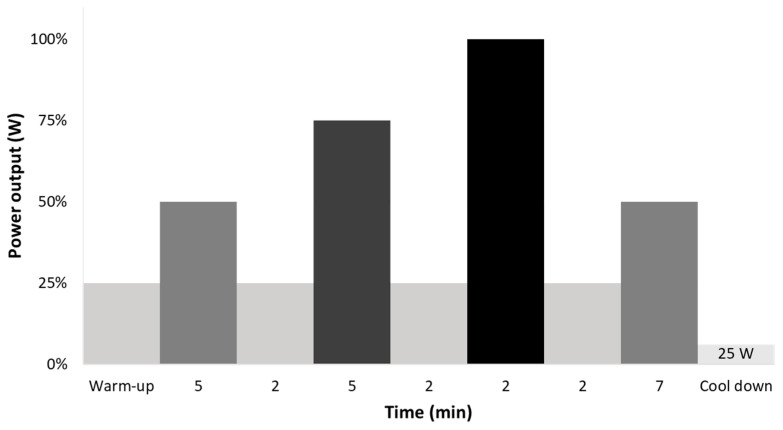
Individualized schematic training session percentages of peak power output.

PPO was used to determine each subject’s training workloads. In the first week (Monday = S1; Tuesday = S2; Wednesday = S3) there were three thirty-minute intermittent training sessions with the fourth day (Thursday = S4) being a sixty-minute session (the thirty-minute session repeated twice). Based on preliminary pilot testing, the session duration was considered adequate to test the effects of fatigue on sRPE in moderately-to-well-trained individuals. Each session started with a five-minute warm-up at 25% PPO followed by 5 min at 50% PPO, 25% PPO for 2 min, 75% PPO for 5 min, 25% PPO for 2 min, 100% PPO for 2 min, 25% PPO for 2 min and 50% of PPO for 7 min, which finished the thirty-minute training session. Interval exercise was chosen because interval training is frequently used to improve the effectiveness of training and make training sessions more interesting. After three days off, the second week consisted of three sixty-minute intermittent training session days (Monday = S5; Tuesday = S6; Wednesday = S7) with the last day (Thursday = S8) being the original thirty-minute workout (e.g., S1). The schematic power output for a thirty-minute session is shown in Figure 1. 

Based on previous work [17], a 50% PPO approximates the ventilatory threshold and a 75% PPO approximates the respiratory compensation threshold. During the two-week training period, the subjects were instructed to “train easily” on days when they did not come to the laboratory. If subjects reported heavy exercise (e.g., intermural sports) when we inquired about their pre-testing training habits, the session was deferred to another time. During training, HR was measured using radiotelemetry (Polar, Electro OY, Kempele, Finland) at rest and at the end of each minute and was expressed as a percent of HR_max_ (%HR_max_). HLa concentration was measured using dry chemistry (Lactate Plus, Nova Biomedical Corporation, Waltham, MA, USA). RPE was measured using the Borg CR-10 scale at 5, 10, 17, 21, and 30 min during the thirty-minute training sessions and at 5, 10, 17, 21, 30, 40, 47, 51 and 60 min during the sixty-minute training sessions. For this study, HR, HLa concentration and RPE were averaged in order to obtain a single mean value for each training session. Further, the HLa/RPE ratio of each training session was calculated as an index of fatigue [18]. Thirty minutes after the completion of the training session, sRPE was obtained by asking “how hard was your workout?” [9,10].

### 2.3. Statistical Analysis

Stata statistical software version 14.1 (Stata-Corp, College Station, TX, USA) was used for statistical analysis. Means, standard deviations (SD) and 95% confidence intervals (95%CI) were calculated for all variables. A multilevel model regression (or hierarchical linear model) was performed to examine the effects of progressive fatigue on subjective and objective training intensity markers. Subjects were considered as the random effect, whereas the training sessions were treated as the fixed effect. The models were fitted using the residual maximum likelihood to account for the small sample. The contrast method was used to test whether the dependent variable (i.e., sRPE, HLa concentration) means of each session were identical. The contrast method tests include ANOVA-style tests of the main effects used to make comparisons against the reference categories (S1, S4 and reverse adjacent training session). Bonferroni post-hoc tests were used for multiple-comparison adjustments across all terms. Non-linear regression analysis was used to analyze the relationship between HLa/RPE ratio and sRPE. The magnitude of correlations was defined by the following criteria: trivial (less than 0.10), small (from 0.10 to 0.29), moderate (from 0.30 to 0.49), large (from 0.50 to 0.69), very large (from 0.70 to 0.89), and almost perfect (from 0.90 to 1.0) [19]. The root-mean-squared error (RMSE) was also calculated for the non-linear regression analysis. Statistical significance was set at *p* < 0.05.

## 3. Results

Descriptive statistics for the subjects are presented in Table 1. 

**Figure 2:**
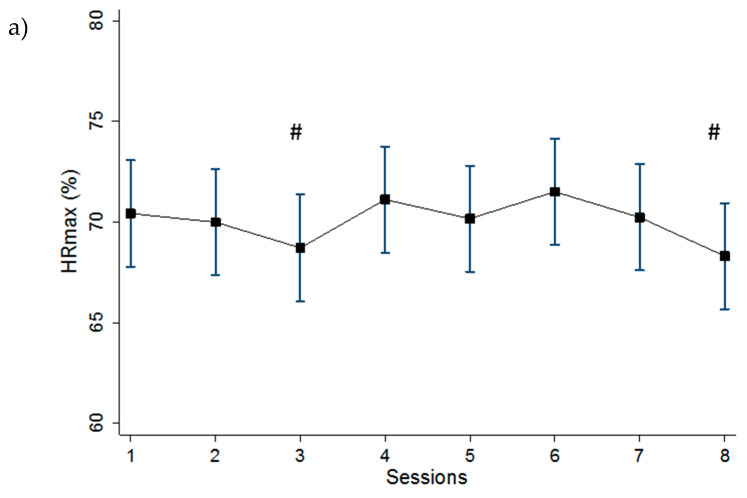

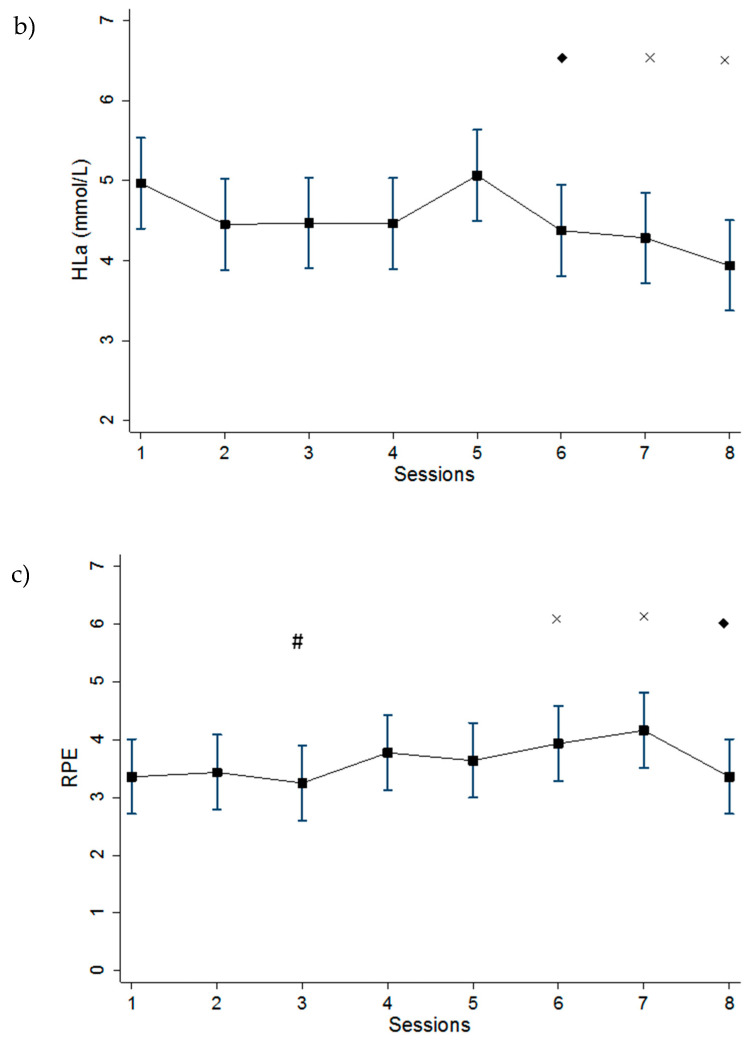
Means and 95% confidence intervals (95%CI) of (**a**) percent of maximal heart rate (%HR_max_), (**b**) blood lactate concentration (HLa) and (**c**) rating of perceived exertion (RPE) across the eight training sessions. ˟: Significantly (*p* < 0.05) different from session 1; #: significantly (*p* < 0.05) different from session 4; ♦: significantly (*p* < 0.05) different from reverse adjacent session.

**Figure 3:**
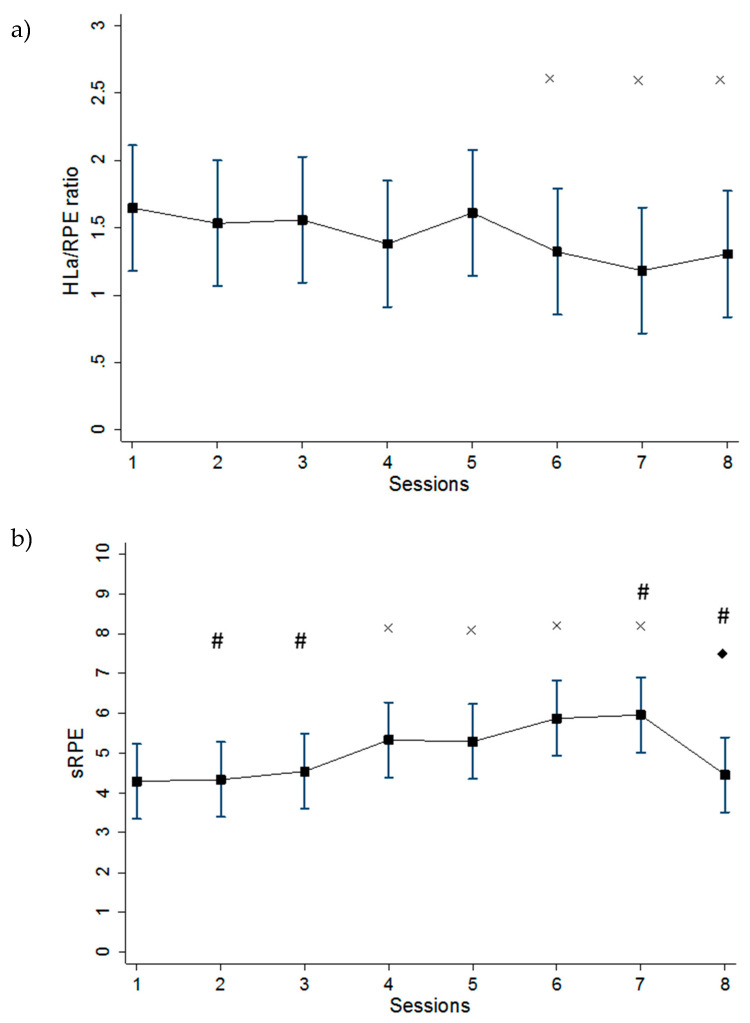
Means and 95% confidence intervals (95%CI) of the (**a**) ratio of blood lactate concentration to ratings of perceived exertion (HLa/RPE ratio) and (**b**) session rating of perceived exertion (sRPE) across the eight training sessions. ˟: Significantly (*p* < 0.05) different from session 1; #: significantly (*p* < 0.05) different from session 4; ♦: significantly (*p* < 0.05) different from reverse adjacent session.

The mixed effects linear regression analysis showed significant differences for %HR_max_ (F_7,77_ = 3.34, *p* = 0.004), HLa concentration (F_7,77_ = 5.04, *p* = 0.0001), average RPE (F_7,77_ = 7.98, *p* < 0.0001), HLa/RPE (F_7,77_ = 4.71, *p* = 0.0002) and sRPE (F_7,77_ = 10.33, *p* < 0.0001) across training sessions. Comparisons after Bonferroni corrections between the training sessions against the reference categories are shown in Figure 2 and Figure 3. 

**Figure 4:**
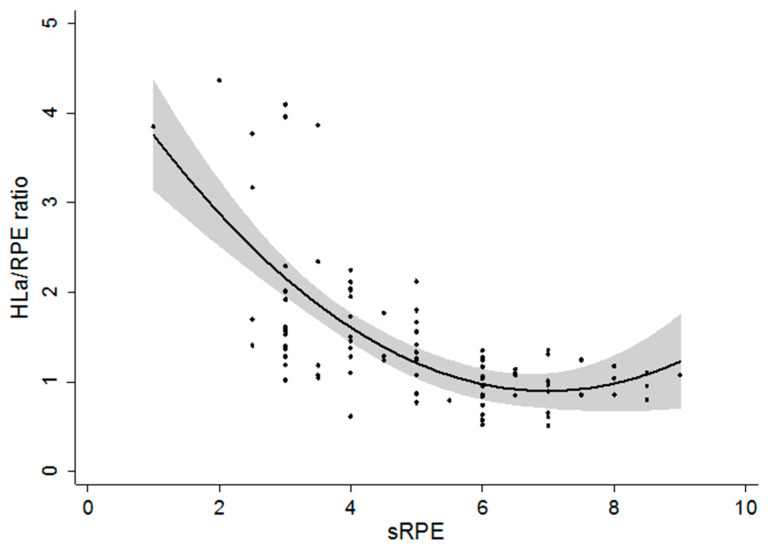
Relationship between the ratio of blood lactate concentration to ratings of perceived exertion (HLa/RPE ratio) and the session rating of perceived exertion (sRPE). The black dots represent all subjects’ training sessions; the black line represents the predicted mean; the grey shaded area represents the 95% confidence interval of the predicted mean.

Non-linear regression analysis revealed a very large negative relationship between HLa/RPE ratio and sRPE ratings for the intermittent training sessions (*r* = −0.70, RMSE = 0.59, *p* < 0.0001) (Figure 4).

## 4. Discussion

The purpose of this study was to examine the effects of progressive fatigue occurring in response to heavier-than-normal training on sRPE. The results of this study demonstrate that, at a constant external training intensity, sRPE increases with session duration and sequential days, which may provide more information on accumulated fatigue, supplementary to information regarding internal training intensity [9,10].

Training session %HR_max_ mean values were relatively constant over the two-week period. However, S3 and S8 were significantly lower than S4. We hypothesize that this difference in training %HR_max_ could be due to the effects of the lengths of the two workouts (thirty-minute versus sixty-minute). It has been demonstrated that the connection between HR responses and training intensity is influenced by several factors, such as duration, frequency, and training status. Previous studies have demonstrated that HR at a fixed submaximal exercise intensity is augmented with increasing bout duration, in presence of overtraining or with a lack of conditioning, but conversely decreases as aerobic fitness improves [20,21]. Lamberts et al. [22] have shown that, under controlled conditions in which the training status does not change, submaximal HR might vary ±7 bpm when the exercise intensity is approximately 90% of HR_max_. In our study, subjects performed intermittent training sessions (25–100% of PPO). Although high intensities were reached momentarily during the training sessions, HR was submaximal and relatively constant throughout the two-week period. Furthermore, the difference between the training session HR ranged from 1 to 5 bpm, which is within the magnitude of day-to-day variation previously suggested [22].

Monitoring HLa concentration is a common method to evaluate responses to training. As the intensity of exercise increases, HLa concentration increases, at least beyond the commonly accepted ‘lactate threshold’. This increase in HLa concentration illustrates a reliance on the glycolytic process, which is the breakdown of glucose or glycogen into lactate [23]. In our study, the accumulated HLa concentration of S7 and S8 were significantly lower than S1. A similar trend in muscle glycogen decrease was found after successive days of heavier-than-normal exertion [5]. The decrease in HLa concentration observed here paralleled the progressive decrease in muscle glycogen. We have shown that an acute increase in training intensity or a workload that is likely to cause acute glycogen depletion typically leads to decreases in HLa concentration at fixed workloads [6]. As glycogen depletion has been thought to contribute to fatigue during high intensity exercise and might be part of the overtraining syndrome [24], we could assume that the decrease in HLa concentration found in our study could be due to the effects of accumulated fatigue present during longer training bouts and during a sequence of longer training bouts. This hypothesis could be further explained by the significant differences found between S5 and S6. In fact, the subjects had 96 h of rest before performing S5 and, therefore, it could be inferred that they had enough time for adequate glycogen resynthesis.

Training RPE mean values were relatively constant over the two-week period, with values ranging from 3.3 to 4.2. To the best of our knowledge, only a few studies have investigated the effects of consecutive match or controlled training days on RPE [25,26]. Gescheit et al. [25] reported no significant differences in RPE during four consecutive days of prolonged tennis match play in trained players. The authors inferred that the player’s RPE remained constant over the four days due to the potential of pacing and tactical modifications (e.g., downregulation of exercise intensity to maintain perceived effort). Haddad et al. [26] investigated the influence of fatigue, stress, muscle soreness and sleep on RPE during submaximal effort. They showed that RPE during a submaximal exercise was not influenced by sleep, stress, fatigue, and delayed onset muscle soreness during a ten-minute standardized submaximal warm-up with young soccer players. However, in our study, we found significant differences between the very last training sessions and the reference sessions. The significant differences found between S6 and S7, with respect to S1, might be due to accumulated fatigue at the end of the two-week period, whereas the significant differences found between S3 versus S4 and S7 versus S8 might be due to the paired effects of weekly accumulated fatigue and the impact of the session duration.

Although the 0–10 RPE scale has been shown to have strong positive correlation with HLa concentration during exercise [11], there is evidence to support the concept that the RPE–HLa relationship is altered during extended cycling at a steady workload [27], and during repeated bouts of exercise [16]. This alteration does not seem to be influenced by recovery time between bouts (up to 3.5h) [28]. Several studies have shown that the HLa/RPE ratio might be considered a useful method to detect the effects of training programs and the occurrence of short-term overreaching [18,29]. Snyder et al. [18] used the HLa/RPE ratio to detect over-reached status in competitive cyclists, by showing that the HLa/RPE ratio decreased for all workloads following two weeks of intensive interval training. Accordingly, our results showed a similar altered relationship between RPE and HLa concentration over the two-week training period. As we found significant differences in HLa/RPE between S6, S7 and S8 versus S1, we infer that the decrease in HLa/RPE could be due to the paired effects of HLa concentration decrease and RPE increase at the end of the two-week training period. Therefore, HLa/RPE could be a useful surrogate for monitoring accumulated fatigue over prolonged periods of training. However, future studies should investigate its consistency and reliability.

Regarding sRPE, the present findings support the hypothesis that sRPE may significantly increase as a longer-than-usual training load progresses. Overall, the sixty-minute intermittent training sessions showed significantly higher sRPE with respect to the thirty-minute sessions. The results support the concept that sRPE reflects information beyond the internal intensity of exercise and whether acutely (during a sixty-minute or thirty-minute workout), or sub-acutely (during 3 consecutive days of higher-than-usual training), this may reflect accumulating fatigue, in addition to exercise intensity. Herman et al. [30] have also shown that sRPE increases after progressive fatigue from continuous bouts of exercise. Fusco et al. [16] have shown that sRPE may provide information about accumulated fatigue during a single prolonged training bout, while other markers of intensity, such as HR and HLa concentration, remained constant. In this study, the workouts were formatted in a fashion that strained the subjects enough during the sixty-minute sessions to elicit a decrease in HLa concentration and an increase in sRPE. Based on our results, it might be assumed that the decrease in HLa concentration, paired with the increase in sRPE, could be a potential indicator that the subjects were unable to replenish their muscle glycogen stores adequately between the hard workouts, especially at the end of the second week. Therefore, sRPE might be a sensitive tool for monitoring the internal training load that provides further information on accumulated fatigue. This hypothesis might be further explained by the significantly large negative relationship between HLa/RPE and sRPE.

Despite the findings of this study, some limits need to be acknowledged. Firstly, we were limited to using HLa concentration and HLa/RPE as surrogate measurements for muscle glycogen concentrations. To get a more accurate depiction of physiological fatigue, it would be beneficial to replicate this study in a setting that allows for the direct measurement of muscle glycogen. Furthermore, the subjects’ diets were not controlled, and consequently, it would be beneficial to carry out other studies with subjects on a specific diet, such as a high carbohydrate intake designed to maintain carbohydrate reserves. Finally, as during the sixty-minute training sessions there was a significant increase in sRPE, paired with a significant decrease in HLa concentration, it is worthwhile to speculate whether the data would likely have more clearly supported our hypothesis if the sixty-minute sessions were extended for a longer time (either acutely or for more days) [31]. Even so, this study provides evidence that sRPE provides information that is more complex than simply providing a marker of exercise intensity. Therefore, future studies are required to explore the effectiveness of sRPE as a simple method for monitoring accumulated fatigue and avoiding inadequate recovery or overtraining.

## 5. Conclusions

In conclusion, the results support the concept that sRPE is a sensitive tool that may detect accumulated fatigue across multiple training days, in addition to being a surrogate marker of exercise intensity. Coaches, health scientists and practitioners without access to HLa concentration measurements may gain insight into accumulated fatigue during periods of increased training by using sRPE in order to avoid inadequate recovery or overtraining. 

## Figures and Tables

**Table 1:** Descriptive characteristics of the subjects (mean ± standard deviation (SD)).

Characteristics	Females (*n* = 6)	Males (*n* = 6)
Age (years)	21.2 ± 3.0	21.2 ± 2.9
Mass (kg)	67.5 ± 8.8	76.8 ± 5.7
Height (cm)	171.0 ± 8.6	176.1 ± 4.1
Peak Power Output (W)	190.5 ± 24.6	258.5 ± 31.0
VO_2peak_ (mL/kg/min)	46.8 ± 2.6	51.8 ± 6.1
